# Right Ventricular–Pulmonary Artery Coupling as a Prognostic Marker in Cardiac Amyloidosis: A Comprehensive Review

**DOI:** 10.3390/life16010109

**Published:** 2026-01-12

**Authors:** Nikolaos Tsiamis, Dimitrios Afendoulis, Christos Tountas, Fotios Toulgaridis, Flora Tsakirian, Sotirios Tsalamandris, Maria Drakopoulou, Kostas Tsioufis, Anastasia Kitsiou, Konstantinos Toutouzas

**Affiliations:** 1 Cardiology Department, Sismanogleio General Hospital, 15126 Athens, Greece; tountasxristos@gmail.com (C.T.); fotistoulgaridis@gmail.com (F.T.); anastasia.kitsiou@gmail.com (A.K.); 2 Unit of Structural and Valvular Heart Diseases, 1st Department of Cardiology, National and Kapodistrian University of Athens (NKUA), ‘Hippokration’ General Hospital of Athens, 11527 Athens, Greece; loratsakirianmed@gmail.com (F.T.); stsalamandris@hotmail.com (S.T.); mdrakopoulou@hotmail.com (M.D.); ktsioufis@gmail.com (K.T.); ktoutouz@gmail.com (K.T.)

**Keywords:** cardiac amyloidosis, RV–PA coupling, TAPSE/PASP, right ventricular function, prognosis, pulmonary hypertension, ATTR, AL amyloidosis

## Abstract

**Background:** Cardiac amyloidosis (CA) is characterized by progressive myocardial infiltration leading to restrictive cardiomyopathy and heart failure. While left ventricular assessment has traditionally dominated prognostic evaluation, right ventricular (RV) dysfunction and RV–pulmonary artery (PA) coupling have emerged as critical determinants of outcomes. **Objectives:** This review synthesizes current evidence on RV–PA coupling as a prognostic marker in cardiac amyloidosis, examining measurement methodologies, prognostic significance, pathophysiological mechanisms, and clinical applications. **Methods:** We comprehensively reviewed the recent literature on RV–PA coupling in CA, focusing on studies published from 2020 to 2025, including both AL and ATTR subtypes. We analyzed data from multicenter cohorts, prospective registries, and validation studies examining the relationship between RV–PA coupling indices and clinical outcomes. **Results:** RV–PA coupling, most commonly assessed using the tricuspid annular plane systolic excursion to pulmonary artery systolic pressure (TAPSE/PASP) ratio, consistently demonstrates strong independent prognostic value for mortality and heart failure outcomes in CA patients. Impaired coupling (TAPSE/PASP < 0.45 mm/mmHg) identifies high-risk patients with hazard ratios ranging from 1.98 to 4.17 for adverse outcomes. In a multicenter cohort of 283 patients, TAPSE/PASP < 0.45 mm/mmHg was independently associated with death or heart failure hospitalization (HR 1.98, 95% CI 1.32–2.96, *p* = 0.001) and significantly improved risk reclassification (NRI 0.46–0.49). In ATTR-specific populations receiving disease-modifying therapy, impaired coupling (TAPSE/PASP ≤ 0.382 mm/mmHg) predicted three-year mortality with an adjusted HR of 2.99. The coupling index provides incremental value over individual RV parameters by accounting for afterload conditions and demonstrates consistent prognostic performance across both AL and ATTR subtypes. **Conclusions:** RV–PA coupling represents a robust, easily obtainable prognostic marker that should be routinely assessed in CA patients for risk stratification and clinical decision-making. The TAPSE/PASP ratio can be calculated from standard echocardiographic examinations without additional cost or time, making it practical for widespread implementation. Future research should focus on standardizing measurement protocols, establishing disease-specific thresholds, evaluating coupling trajectories with novel therapies, and integrating coupling assessment into staging systems and management algorithms. The strong prognostic signal, pathophysiological relevance, and ease of measurement position RV–PA coupling as an essential component of comprehensive cardiac amyloidosis evaluation.

## 1. Introduction

### 1.1. Cardiac Amyloidosis and Unmet Prognostic Needs

Cardiac amyloidosis (CA) is a progressive infiltrative cardiomyopathy caused by extracellular deposition of misfolded protein fibrils within the myocardium, resulting in restrictive physiology, heart failure, and increased mortality [1]. The two predominant etiologies—immunoglobulin light-chain (AL) amyloidosis and transthyretin-related (ATTR) amyloidosis—account for most of the cardiac involvement [2,3]. Contemporary cohort studies have highlighted the clinical impact of CA, including the high prevalence of advanced heart failure and adverse outcomes across both AL and ATTR subtypes [2,3,4]. Despite advances in diagnostic pathways, prognosis remains guarded, particularly in advanced disease, underscoring the need for accurate and clinically meaningful risk stratification.

In recent years, the therapeutic landscape has evolved, especially for ATTR cardiac amyloidosis. Tafamidis has demonstrated clinical benefit in ATTR amyloid cardiomyopathy, supporting earlier diagnosis and refined prognostic evaluation in the modern treatment era [5]. In parallel, contemporary real-world cohorts receiving transthyretin stabilizers continue to show that meaningful risk stratification remains necessary to guide monitoring and management [6].

Traditionally, prognostic assessment in CA has relied on biomarker-based staging systems and left ventricular (LV) structural and functional indices, from the basis of widely used staging models in both AL and ATTR amyloidosis, and retains strong prognostic value [7,8,9]. However, these approaches predominantly reflect LV involvement and may not fully capture the complex biventricular pathophysiology of the disease.

### 1.2. Right Ventricular Involvement and the Rationale for RV–PA Coupling

Cardiac amyloidosis is increasingly recognized as a biventricular disease, with right ventricular (RV) involvement occurring through multiple mechanisms, including direct amyloid infiltration of the RV myocardium, elevated left-sided filling pressures, secondary pulmonary hypertension, ventricular interdependence, and pericardial constraint [10,11,12]. RV dysfunction is common in advanced clinical outcomes, providing prognostic information beyond LV-focused parameters [12,13].

Assessment of RVfunction remains challenging due to the complex geometry of the right ventricle and its marked sensitivity to loading conditions. Conventional echocardiographic parameters, such as tricuspid annular plane systolic excursion (TAPSE), RV fractional area change, and RV longitudinal strain, primarily reflect RV contractility but do not account for pulmonary vascular afterload [14,15]. Right ventricular–pulmonary artery (RV–PA) coupling integrates RV contractile performance with afterload, offering a more physiologically relevant assessment of RV function and its ability to adapt to increased pulmonary pressures [16,17].

Non-invasive estimation of RV–PA coupling using the TAPSE-to-pulmonary artery systolic pressure (PASP) ratio has emerged as a practical and widely applicable surrogate of ventriculo-arterial coupling. This index can be derived from standard transthoracic echocardiography without additional imaging time or cost and demonstrates reasonable correlation with invasively measured coupling parameters [18,19,20,21]. Importantly, multiple studies have demonstrated that impaired RV–PA coupling is a strong independent predictor of mortality and heart failure outcomes in CA across both AL and ATTR subtypes [2,3,4], including in contemporary ATTR populations receiving disease-modifying therapy [6].

### 1.3. Aim and Scope of the Present Review

Given the growing body of evidence supporting the prognostic relevance of RV–PA coupling and the expanding therapeutic landscape of cardiac amyloidosis, a comprehensive synthesis of current data is timely. The aim of this review is to summarize the physiological basis of RV–PA coupling, critically evaluate available measurement methodologies, synthesize prognostic evidence across AL and ATTR cardiac amyloidosis, and discuss clinical applications, limitations, and future research directions. By integrating current knowledge, this review stratifies and highlights its potential utility in routine clinical practice.

## 2. Physiology and Pathophysiology of RV–PA Coupling

### 2.1. Normal RV–PA Coupling Physiology

The right ventricle and pulmonary arterial system function as a coupled unit, with optimal efficiency achieved when RV contractility matches pulmonary vascular load. In healthy individuals, the RV operates at low afterload with high compliance, maintaining an E_es_/E_a_ ratio of approximately 1.5–2.0, indicating contractile reserve. This coupling ensures efficient energy transfer and optimal stroke volume with minimal oxygen consumption.

The physiological principle underlying ventriculo-arterial coupling is that maximal mechanical efficiency occurs when ventricular elastance matches arterial elastance. The RV, with its thin-walled crescent-shaped and predominantly longitudinal fiber orientation, is exquisitely sensitive to changes in afterload. Under normal conditions, the low-resistance, high-compliance pulmonary circulation allows the RV to maintain adequate cardiac output with minimal oxygen consumption and energy expenditure.

### 2.2. Progression from Coupling to Uncoupling

RV–PA coupling deterioration in CA follows a progressive pattern that can be conceptualized in the following stages:

Stage 1 (Compensated): Normal TAPSE/PASP despite early infiltration; RV compensates through increased contractility and recruitment of contractile reserve. Patients may be asymptomatic or have minimal symptoms. Biomarkers may be mildly elevated.

Stage 2 (Early Uncoupling): Borderline TAPSE/PASP (0.40–0.50 mm/mmHg); RV contractile reserve diminishes. Patients typically have mild to moderate symptoms (NYHA class II). Biomarkers are moderately elevated. Exercise capacity begins to decline.

Stage 3 (Manifest Uncoupling): TAPSE/PASP < 0.40 mm/mmHg; RV unable to maintain output against elevated afterload. Patients have moderate to severe symptoms (NYHA class III–IV). Biomarkers are significantly elevated. Exercise capacity is markedly reduced.

Stage 4 (Decompensated): Severe uncoupling with clinical right heart failure, elevated central venous pressure, and end-organ dysfunction (hepatic congestion, renal dysfunction, peripheral edema). Patients are typically NYHA class IV with symptoms at rest. Prognosis is poor without advanced interventions.

This progression is not necessarily linear and may be accelerated by intercurrent illness, atrial fibrillation, or other comorbidities. Some patients may remain in compensated stages for extended periods, while others demonstrate rapid progression. Understanding this natural history is important for the timing of therapeutic interventions and prognostic counseling.

## 3. Methods

### 3.1. Research Strategy

The current review was reported in accordance with the preferred reporting items for systematic reviews (PRISMA). A comprehensive search of the literature was conducted through the PubMed and Google Scholar databases from inception to December 2025, to identify relevant studies. The search strategy included the following keywords: ‘Right Ventricular and Pulmonary artery coupling’, ‘RV–PA Coupling’and ‘Amyloidosis’. Boolean research terms included [“RV-PA Coupling”], [“Right Ventricular-Pulmonary artery coupling’’] AND [“Amyloidosis’’]. Synonyms and equivalent terms for these keywords were also included, as well as reference lists of the articles included in the review, which were screened for additional citations to ensure broad research.

### 3.2. Eligibility Criteria, Screening, and Data Extraction

Studies were eligible for inclusion if they focused on amyloidosis and right ventricular–pulmonary artery coupling. This included the correlation between amyloidosis and RV–PA coupling in terms of prognosis or treatment strategies. Both AL-amyloidosis and ATTR-amyloidosis were included in our review. Exclusion criteria included abstracts, editorials, case reports, and animal studies, or non-English articles. Moreover, any articles not relevant to the correlation between RV–PA coupling and amyloidosis regarding prognostic implications, treatment, or management options were excluded from our review. After removing duplicate articles, all available articles were screened for title and abstract relevance. Furthermore, the full texts of potentially eligible studies were assessed by the same reviewers. Key data were extracted by the reviewers, focusing on study and population characteristics and the correlation of RV–PA coupling with treatment or prognosis of amyloidosis.

#### 3.2.1. Quality Assessment

During data extraction, significant methodological heterogeneity was noted in the reporting of the primary prognostic measure, the TAPSE/PASP ratio. Two studies reported the Hazard Ratio (HR) as a continuous variable, quantifying the risk change per unit increase in the TAPSE/PASP ratio. Two studies reported the HR as a dichotomous variable, comparing the risk of a “low” TAPSE/PASP group against a “high” group based on a predetermined cut-off. Despite heterogeneity in study outcomes, amyloidosis subtypes, and cut-off values for the TAPSE/PASP ratio, all studies consistently demonstrated that RV–PA uncoupling has a significant prognostic impact in patients with amyloidosis. While statistical heterogeneity and wide Hazard Ratio (HR) ranges constitute a limitation for prognostic modeling, they do not undermine the main findings of this review.

#### 3.2.2. Results and Discussion

According to our database research, 72 articles were initially screened, and 39 duplicate articles were excluded. Eventually, 13 articles were evaluated by our reviewers, and 6 studies were included in our review (Table 1). The type of studies included were four retrospective and two prospective, with a total population of 1197 patients. The PRISMA flowchart for our selection process is presented in the Supplementary File.

## 4. Prognostic Evidence in Cardiac Amyloidosis

### 4.1. ATTR Cardiac Amyloidosis

In transthyretin cardiac amyloidosis (ATTR-CA), right ventricular–pulmonary artery (RV–PA) coupling has emerged as a robust prognostic marker. Evidence from contemporary ATTR cohorts consistently demonstrates that impaired RV–PA coupling, most commonly assessed using the TAPSE/PASP ratio, is independently associated with increased mortality and adverse clinical outcomes.

In a large ATTR-specific cohort, Schwarting et al. evaluated RV–PA coupling in patients with ATTR cardiac amyloidosis receiving transthyretin stabilizer therapy and identified a TAPSE/PASP cutoff of ≤0.382 mm/mmHg as optimal for mortality prediction. During follow-up, impaired RV–PA coupling remained an independent predictor of all-cause mortality after adjustment for established clinical, biomarker, and echocardiographic prognostic variables [6]. Importantly, the prognostic value of RV–PA coupling persisted despite treatment with tafamidis, indicating that RV–PA uncoupling reflects advanced disease severity and hemodynamic compromise beyond the effects of disease-modifying therapy [5,6].

In addition to mortality, RV–PA coupling has been linked to functional status in ATTR-CA. Reduced TAPSE/PASP ratios have been associated with worse New York Heart Association functional class and impaired exercise capacity, underscoring the close relationship between RV–PA uncoupling and symptomatic burden in this population.

Sinigiani et al. (2025) demonstrated in 100 wtATTR-CM patients that RV–PA uncoupling indices (TAPSE/sPAP, RVFWLS/sPAP) were strong, independent predictors of mortality and HF hospitalization (HR 0.04–0.07). Specific cut-offs (TAPSE/sPAP ≤ 0.45 mm/mmHg and RVFWLS/sPAP ≤ 0.46%/mmHg) provided incremental prognostic value over standard RV parameters, significantly stratifying risk even in early-stage disease [23].

Overall, available evidence indicates that RV–PA coupling provides clinically relevant prognostic and functional information in ATTR cardiac amyloidosis, including in patients receiving contemporary disease-modifying therapies.

### 4.2. AL Cardiac Amyloidosis

In immunoglobulin light-chain cardiac amyloidosis (AL-CA), right ventricular–pulmonary artery (RV–PA) coupling demonstrates particularly strong prognostic value, reflecting the aggressive clinical course and rapid hemodynamic deterioration characteristic of this subtype. Several studies have shown that impaired RV–PA coupling is associated with markedly worse short-term outcomes in AL-CA.

Yu et al. evaluated RV–PA coupling in a cohort of patients with AL cardiac amyloidosis and demonstrated that a reduced TAPSE/PASP ratio (<0.47 mm/mmHg) was a strong predictor of 6-month all-cause mortality, with good discriminatory performance. Patients with impaired coupling experienced significantly higher early mortality, highlighting the utility of RV–PA coupling for short-term risk stratification in AL-CA [22].

Importantly, the prognostic impact of RV–PA uncoupling in AL cardiac amyloidosis appears to be amplified when combined with markers of advanced disease severity. In mixed AL cohorts, patients with impaired RV–PA coupling and low systolic blood pressure represent a particularly high-risk subgroup with markedly reduced survival, emphasizing the interaction between RV dysfunction, afterload mismatch, and systemic hemodynamic compromise [3].

Overall, available evidence indicates that RV–PA coupling provides powerful early prognostic information in AL cardiac amyloidosis. Given the rapid disease progression and high short-term mortality associated with AL-CA, assessment of RV–PA coupling may be particularly valuable for early risk stratification and clinical decision-making.

### 4.3. Mixed CA Populations

Several studies evaluating mixed populations of patients with cardiac amyloidosis, including both AL and ATTR subtypes, have consistently demonstrated the prognostic relevance of right ventricular–pulmonary artery (RV–PA) coupling. These analyses provide important evidence that RV–PA coupling retains prognostic value across the spectrum of cardiac amyloidosis, independent of amyloid subtype.

In a large multicenter cohort including patients with both AL and ATTR cardiac amyloidosis, Tomasoni et al. assessed RV–PA coupling using the TAPSE/PASP ratio and demonstrated that impaired coupling (<0.45 mm/mmHg) was independently associated with a higher risk of all-cause mortality and heart failure hospitalization. Importantly, the TAPSE/PASP ratio provided incremental prognostic value beyond established biomarkers and conventional echocardiographic parameters, significantly improving risk reclassification and discrimination metrics [2].

Similar findings have been reported in other mixed-population studies. Maccallini et al. confirmed the prognostic significance of the TAPSE/PASP ratio in a contemporary cohort of patients with cardiac amyloidosis, demonstrating that RV–PA uncoupling was associated with adverse outcomes regardless of amyloid subtype [4]. In addition, Palmiero et al. reported that RV–PA uncoupling was common in mixed cardiac amyloidosis populations and was associated with more advanced clinical disease and worse prognosis [3].

Overall, evidence from mixed cardiac amyloidosis cohorts supports RV–PA coupling as a robust and broadly applicable prognostic marker. The consistency of its predictive value across AL and ATTR subtypes highlights its utility as an integrative measure of right heart–pulmonary vascular interaction in cardiac amyloidosis.

### 4.4. Heart Failure Outcomes and Functional Status

Beyond mortality, right ventricular–pulmonary artery (RV–PA) coupling has demonstrated a strong association with heart failure-related morbidity and functional impairment in patients with cardiac amyloidosis. Across multiple cohorts, impaired RV–PA coupling has been consistently linked to higher rates of heart failure hospitalization, reflecting advanced hemodynamic compromise and right-sided dysfunction.

Patients with reduced TAPSE/PASP ratios exhibit a significantly worse New York Heart Association functional class and reduced exercise tolerance. Studies in both ATTR and mixed cardiac amyloidosis populations have shown that RV–PA uncoupling correlates with lower six-minute walk distance and impaired cardiopulmonary exercise performance, highlighting the close relationship between RV–PA coupling, pulmonary vascular load, and symptomatic burden.

Importantly, the association between RV–PA coupling and heart failure outcomes appears independent of left ventricular systolic function and traditional biomarkers. This finding supports the concept that RV–PA uncoupling captures downstream pathophysiological consequences of cardiac amyloidosis that are not fully reflected by conventional prognostic markers. As such, RV–PA coupling provides clinically meaningful information regarding both disease severity and functional limitation in patients with cardiac amyloidosis. Multivariate analysis of biomarkers in amyloidosis, such as NT-proBNP and troponin, and their correlation with disease progression was not performed in the context of our review, as it would be more suitable for a meta-analysis, and it is already well established in the literature. As it is highlighted, RV–PA coupling presents a factor of disease severity and progression beyond biomarkers and should be evaluated along with these parameters and symptoms for a more holistic understanding of disease severity [24,25,26]. Moreover, RV–PA coupling could be an objective parameter for assessing the risk of the appearance of symptoms of right ventricular, and could thus be used in clinical practice for the evaluation of the duration of follow-up, intensification of treatment, and assessment of disease progression [24,25,26,27,28].

## 5. Clinical Applications and Risk Stratification

### 5.1. Initial Diagnostic Evaluation

The TAPSE/PASP thresholds used in the included studies were heterogeneous and not guideline-derived. Based on Pulmonary Hypertension guidelines, a threshold of 0.45 is suggested as a reference value. As demonstrated, a reduction in the TAPSE/PASP ratio below specific thresholds—specifically below 0.38, which defines uncoupling—was associated with worst outcomes and progression to right ventricular failure and/or appearance of symptoms.

### 5.2. Serial Monitoring and Disease Progression

Recommended echocardiographic reassessment intervals include every 6–12 months for ATTR-CA on tafamidis, every 3–6 months for AL-CA during treatment, and prompt reassessment with symptomatic worsening. Coupling trajectory provides important prognostic information: stable coupling suggests disease control, improving coupling indicates favorable treatment response, declining coupling signals disease progression, and threshold crossing warrants clinical action.

### 5.3. Recommendation for Clinical Practice

These results are derived from observational data and are primarily hypothesis-generating in nature; therefore, they should not be interpreted as established clinical guidelines. Based on current evidence, we recommend the following: (1) routine TAPSE/PASP assessment and reporting in all CA patients; (2) risk stratification using established thresholds; (3) serial monitoring at appropriate intervals; (4) integrated approach with biomarkers and clinical parameters; (5) consideration of coupling status in clinical decisions; and (6) quality assurance through standardized protocols.

## 6. Future Directions and Research Priorities

### 6.1. Validation and Standardization

Future research priorities include multicenter prospective registries with standardized imaging protocols, external validation of published cutoffs, population-specific threshold determination, and reproducibility studies. Consensus guidelines are needed to establish measurement standardization, evidence-based threshold recommendations, and clinical integration protocols.

### 6.2. Therapeutic Implications

Further studies are needed to evaluate changes in RV–PA coupling in response to ATTR therapies (tafamidis, patisiran, vutrisiran) and AL therapies, including chemotherapy and novel antibodies. Randomized controlled trials should assess coupling-guided versus standard care, optimal treatment intensification strategies, and targeted interventions such as pulmonary vasodilators and diuretic optimization.

### 6.3. Advanced Imaging Integration

Multimodality approaches should integrate echocardiography with cardiac magnetic resonance, nuclear imaging, and computed tomography to develop multiparametric risk models. Novel imaging biomarkers, including RV myocardial work, four-dimensional flow MRI, and strain rate imaging, require validation in cardiac amyloidosis populations.

### 6.4. Mechanistic Investigations

Mechanistic studies should elucidate the cellular and molecular effects of amyloid deposition on myocardial function, microvascular dysfunction, and fibrotic remodeling. Invasive hemodynamic assessments and exercise testing may further clarify disease progression and natural history. Biomarker discovery efforts should aim to identify novel circulating markers and imaging–biomarker combinations.

### 6.5. Implementation Science

Implementation research should study barriers and facilitators of adoption, develop educational interventions, and optimize workflow integration. Health economics analyses should evaluate cost-effectiveness, resource utilization impact, and value-based care metrics. Global health perspectives must address access and equity in resource-limited settings.

## 7. Clinical Recommendations and Best Practices

### 7.1. Routine Assessment Protocol

At initial diagnosis, a comprehensive echocardiographic evaluation should be performed, including standard two-dimensional and Doppler assessment, TAPSE measurement, tricuspid regurgitation velocity acquisition, inferior vena cava assessment, and TAPSE/PASP calculation. Right ventricular strain analysis may be included when available. Findings should be integrated with clinical symptoms, biomarkers, staging systems, and baseline risk stratification.

For serial monitoring, follow-up echocardiography is recommended every 6–12 months for ATTR cardiac amyloidosis receiving therapy and every 3–6 months for AL cardiac amyloidosis during active treatment, with annual reassessment in stable disease and prompt evaluation in cases of clinical deterioration. Longitudinal assessment should use consistent methodology, trend values over time, identify significant changes (>20%), and correlate findings with clinical status [29,30,31].

### 7.2. Integration into Clinical Practice

**Figure 1:**
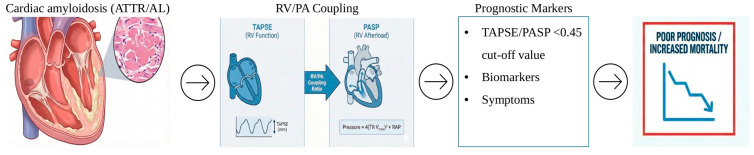
Central illustration.

RV–PA coupling should be interpreted in conjunction with clinical status, biomarkers, and left ventricular parameters rather than as a stand-alone metric. Risk categories derived from TAPSE/PASP thresholds may support clinical decision-making, monitoring frequency, and patient counseling, but should be applied within a comprehensive, individualized assessment framework. Symptoms of right ventricular failure are closely related to impairment of right ventricular functional parameters. Reduced right ventricular systolic function, reflected by decreased right ventricular ejection fraction, low tricuspid annular plane systolic excursion (TAPSE), reduced fractional area change, or impaired longitudinal strain, leads to diminished forward blood flow to the pulmonary circulation, resulting in fatigue, exercise intolerance, and worsening dyspnea. Abnormal right ventricular diastolic function causes elevated filling pressures and impaired ventricular relaxation, which clinically translates into systemic venous congestion manifested by peripheral edema, hepatomegaly, ascites, and jugular venous distension. In addition, impaired coupling between the right ventricle and pulmonary artery, often seen in pulmonary hypertension, limits the ability of the right ventricle to adapt to increased afterload and is associated with severe exercise intolerance and progression of symptoms. The presence of significant tricuspid regurgitation further exacerbates right ventricular volume overload and venous congestion, intensifying clinical signs and symptoms of right-sided heart failure. This clearly indicates that symptom severity should always be taken into consideration as an independent risk factor when evaluating the prognosis of patients with diseases causing pulmonary hypertension and right ventricular failure, such as amyloidosis, along with biomarkers and imaging-measured parameters. (Figure 1) The integration of RV–PA coupling assessment into routine cardiac amyloidosis care offers several important clinical benefits: enhanced risk assessment beyond current staging systems, treatment monitoring for disease progression and therapeutic response, efficient resource allocation with objective risk stratification, and informed decision-making supported by objective data for treatment options and end-of-life planning [30,31].

Right ventricular–pulmonary artery coupling represents an important advancement in prognostic assessment for cardiac amyloidosis, combining physiological relevance with practical clinical applicability. Its strong prognostic value and ease of measurement support incorporation into comprehensive patient evaluation. Ongoing research and standardization efforts are expected to further define its role in personalized risk stratification and management [31].

### 7.3. Quality Assurance

Standardized technical protocols should be implemented to ensure appropriate transducer positioning, adequate image quality, and averaging of multiple measurements. Training and certification programs should include sonographer education, physician interpretation training, competency assessment, and continuing education. Reporting standards should include TAPSE, tricuspid regurgitation velocity and PASP, right atrial pressure estimation method, TAPSE/PASP ratio, risk category, and comparison with prior studies.

## 8. Conclusions

Right ventricular–pulmonary artery (RV–PA) coupling, assessed non-invasively using the TAPSE/PASP ratio, has emerged as a robust and clinically relevant prognostic marker in cardiac amyloidosis. Available evidence consistently demonstrates that impaired RV–PA coupling independently predicts mortality and heart failure outcomes across both AL and ATTR subtypes.

RV–PA coupling provides incremental prognostic value beyond biomarkers, left ventricular function, and conventional right ventricular parameters by integrating right ventricular contractility with pulmonary vascular afterload. The TAPSE/PASP ratio can be readily derived from standard echocardiographic examinations without additional cost, time, or specialized equipment. Pathophysiologically, RV–PA uncoupling reflects the combined effects of amyloid infiltration, secondary pulmonary hypertension, and progressive hemodynamic deterioration. Established thresholds, particularly <0.45 mm/mmHg, enable stratification of patients into clinically meaningful risk categories. Right ventricular–pulmonary artery coupling represents an important advancement in prognostic assessment for cardiac amyloidosis, combining physiological relevance with practical clinical applicability. Its strong prognostic value and ease of measurement support incorporation into comprehensive patient evaluation. Ongoing research and standardization efforts are expected to further define its role in personalized risk stratification and management.

### Limitations of the Review

Some limitations should be acknowledged when interpreting the findings summarized in this review. First, the available evidence on right ventricular–pulmonary artery (RV–PA) coupling in cardiac amyloidosis is derived predominantly from observational retrospective studies, which limits causal interference [2,3,4,6].

Second, there is substantial heterogeneity in coupling definitions, cutoff values, study populations, and clinical endpoints across published studies. Reported TAPSE/PASP thresholds vary depending on amyloid subtype, disease stage, follow-up duration, and outcome of interest, which limits the generalizability of a single universal cutoff for clinical practice [2,3,4,22].

Third, most studies assessing RV–PA coupling rely on echocardiographic estimation of pulmonary artery systolic pressure, which is subject to measurement variability related to tricuspid regurgitation signal quality and right atrial pressure estimation, as described in the manuscript [32]. TAPSE is angle-dependent and reflects primarily longitudinal RV function, and therefore, the TAPSE/PASP ratio represents a simplified surrogate of complex RV–PA interaction [16,17,29].

Finally, data on longitudinal changes in RV–PA coupling in response to disease-modifying therapies remain limited. Although RV–PA coupling retains prognostic significance in treated ATTR cohorts, it is unclear whether improvement in coupling translates into better clinical outcomes [6]. Additionally, most available evidence originates from specialized referral centers, potentially limiting applicability to broader community populations [2,3,4,29].

## Figures and Tables

**Table 1:** Prognostic studies evaluating right ventricular–pulmonary artery coupling in cardiac amyloidosis.

Study		Population	N	Coupling Index	Cut-Off (mm/mmHg)	Endpoint	Key Findings
Tomasoni et al. [2]	Retrospective	AL + ATTR	283	TAPSE/PASP	<0.45 mm/mmHg	All-cause mortality or HF hospitalization	Impaired RV–PA coupling independently predicted adverse outcomes and significantly improved risk reclassification and LV parameters
Macallini et al. [4]	Prospective	AL+ ATTR	233	TAPSE/PASP	Study-specific	All-cause mortality	RV–PA uncoupling was associated with a worse prognosis regardless of amyloid subtype
Schwarting et al. [6]	Retrospective	ATTR	418	TAPSE/PASP	≤0.382	All-cause mortality	Impaired coupling independently predicted 3-year mortality despite transthyretin stabilizer therapy
Yu et al. [22]	Retrospective	AL	71	TAPSE/PASP	<0.47 mm/mmHg	6-month all-cause mortality	Reduced TAPSE/PASP demonstrated strong short-term prognostic value in AL cardiac amyloidosis
Palmiero et al. [3]	Prospective	AL+ ATTR	92	TAPSE/PASP	Not specified	Composite clinical outcomes	RV–PA uncoupling was common and associated with advanced disease severity and poorer outcomes
Sinigiani et al. [23]	Retrospective	wtATTR	100	TAPSE/PASP	Continuous predictor	All-cause mortality and Heart Failure Hospitalization	RV–PA uncoupling emerged as an early and strong predictor of outcome, being independently associated with the risk of HF hospitalization or all—cause death

## Data Availability

The original contributions presented in this study are included in the article/Supplementary Material. Further inquiries can be directed to the corresponding authors.

## Supplementary Materials

The following supporting information can be downloaded at: https://www.mdpi.com/article/10.3390/life16010109/s1, PRISMA flowchart is presented as a supplementary figure.
