# Supplementary figures and images for: Right Ventricular–Pulmonary Artery Coupling as a Prognostic Marker in Cardiac Amyloidosis: A Comprehensive Review
Source: Life (Basel). 2026 Jan 12;16(1):109. doi: 10.3390/life16010109 (PMC12843239; doi:10.3390/life16010109)

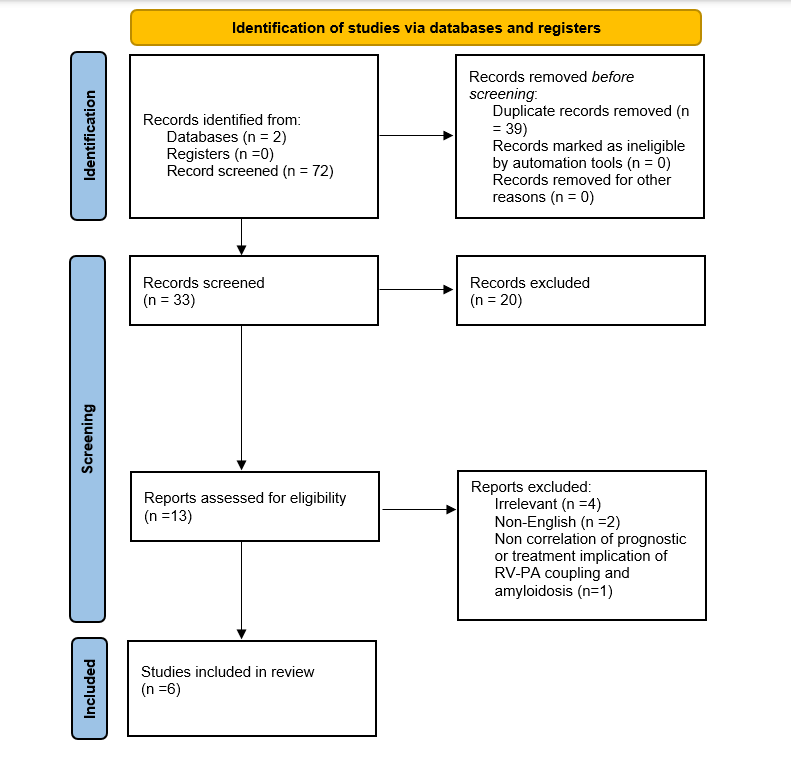

Supplement: Supplementary file 1 [file life-16-00109-s001.zip › life-4061603-supplementary.png]
